# Long-term outcome of perimembranous VSD closure using the Nit-Occlud® Lê VSD coil system

**DOI:** 10.1007/s00392-020-01750-6

**Published:** 2020-10-31

**Authors:** Rainer Kozlik-Feldmann, Avraham Lorber, Horst Sievert, Peter Ewert, Christian Jux, Götz C. Müller, Robert Dalla Pozza, Mustafa Yigitbasi, Dietmar Schranz, Angelika Lindinger, Omar Galal, Thomas Meinertz

**Affiliations:** 1 Department of Pediatric Cardiology, University Heart and Vascular Center, Hamburg, Germany; 2 Department of Pediatric Cardiology and Adults With Congenital Heart Disease, Faculty of Medicine, Technion, Meyer Children’s Hospital of Haifa, Rambam Medical Center, Haifa, Israel; 3 CardioVascular Center Frankfurt, Frankfurt, Germany; 4 Anglia Ruskin University, Chelmsford, UK; 5 Department of Pediatric Cardiology and Congenital Heart Defects, German Heart Center Munich, Munich, Germany; 6 Department of Pediatric Cardiology and Congenital Heart Defects, University Hospital Giessen and Marburg, Giessen, Germany; 7 Department of Pediatric Cardiology and Pediatric Intensive Care, Grosshadern Medical Center, University of Munich, Munich, Germany; 8 Department of Congenital Heart Defects-Pediatric Cardiology, German Heart Center Berlin, Berlin, Germany; 9 Member of the Data Safety and Monitoring Board, Westpfalz-Klinikum, Pediatric Cardiology, Kaiserslautern, Germany; 10 Member of the Data Safety and Monitoring Board, King Faisal Specialist Hospital and RC, Pediatric Cardiology, Jeddah, Saudi Arabia; 11 Head of Clinical Trial and Member of the Data Safety and Monitoring Board, Cardiological-Internal Practice, Hamburg, Germany

**Keywords:** Ventricular septal defect, Interventional therapy, Admission trial, VSD coil system, Five-year follow-up

## Abstract

**Objective:**

This study presents data from the admission trial to show the feasibility, safety and effectiveness of the Nit-Occlud® Lê VSD in the treatment of perimembranous ventricular septal defects with an aneurysmal configuration and a diameter up to 8 mm.

**Background:**

The majority of ventricular septal defects (VSD) are still closed surgically, while a less invasive transcatheter treatment by closure devices is available. Device-based closure is reported to be associated with the risk of complete atrio-ventricular block, especially with double-disc devices in perimembranous defects.

**Methods:**

In six tertiary centers in Germany and Israel, an interventional closure of a periembranous VSD was attempted in 88 patients using the Nit-Occlud® Lê VSD.

**Results:**

The interventional VSD closure was performed in 85 patients. Patients had a median age of 8.0 (2–65) years and a median body weight of 26.7 (10–109) kg. A complete closure of the defects was achieved in 85.4% 2 weeks after device implantation, in 88.9% after three months and in 98.6% at the 5-year follow-up. There was no incidence of death during the study nor did any patient suffer of permanent atrio-ventricular block of higher degree. Serious adverse events, by definition, are potentially life-threatening or require surgery to correct, while major serious events require medical or transcatheter intervention to correct. The study results exhibit a serious adverse event rate of 3.5% (3/85 patients) and a major adverse event rate of 5.9% (5/85 patients).

**Conclusion:**

The Nit-Occlud® Lê VSD coil offers the possibility of an effective and safe approach in patients with aneurysmal perimembranous ventricular septal defects.

## Introduction

The isolated ventricular septal defect (VSD) is the most common congenital heart defect with an incidence in one meta-analysis of 3.5 per 100 births and in some studies with a neonatal rate of more than 50% of all congenital heart defects [12, 18]. These defects cover a wide anatomical spectrum and, about 50% of VSDs are periembranous (pVSD). 77% of patients are asymptomatic [26].

**Fig. 1:**
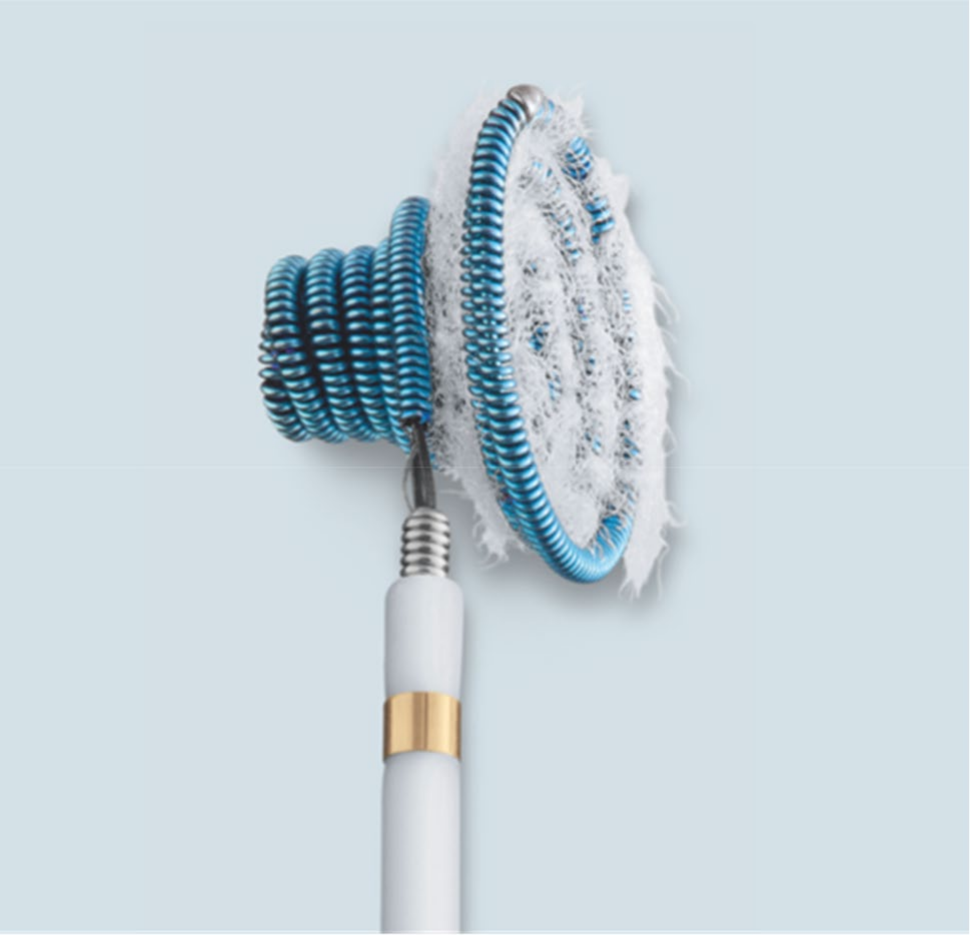
Close-up image of the Nit Occlud® Lê VSD coil. The device configures as larger left-sided cone with reinforced and Dacron fibered distal coil loops and a smaller right-side cone that configures over the left cone

The majority of symptomatic VSDs are still closed surgically. In the last decade, perimembranous and particularly aneurysmal defects increasingly have been occluded in interventional procedures [11, 15, 19, 24]. While most of the devices used for interventional closure are mesh, wire-based double discs, the pfm Nit-Occlud® Lê VSD device has a reinforced coil configuration with Dacron fibers (Fig. 1). The Nit-Occlud® Lê VSD coil received the CE mark in August 2010 and is designed especially for closure of ventricular septal defects located in the perimembranous part of the interventricular septum (in particular those with aneurysmal configuration) and in the muscular septum. We present an intention-to-treat analysis of the multicenter clinical admission trial for the evaluation of the Nit-Occlud Lê VSD coil in the interventional treatment of pVSDs up to 8 mm.

## Methods

### Study objectives

The aim of this study was to demonstrate feasibility, efficacy and safety as well as long-term outcome of VSD closure with the pfm coil system. The presented data are based on a prospective, multicenter and non-randomized clinical trial of the Nit-Occlud® Lê VSD coil sponsored by pfm medical ag (Cologne, Germany). The study was registered at ClinicalTrials.gov on October 20, 2006 with the identifier NCT00390702 as an admission trial of this device. All data generated during this clinical investigation were recorded on electronic CRFs. Internet-based data entry was completed by the clinical investigator or the authorized site coordinator.

Inclusion criteria:

(I) peri-membranous location of the VSD
(II) presence of aneurysmal configuration of the VSD
(III) distance between the rim of the VSD and the aortic annulus of at least 3.0 mm
(IV) diameter of the VSD less than 8 mm in any plane (measured during late diastole by 2-D echocardiography, preferably in apical 5-chamber view)
(V) signs of left atrial or ventricular volume overload (2 standard deviations greater than normal) and/or a shunt fraction (Q_p_/Q_s_) ≥ 1.5 (measured by catheterization) present
(VI) patient age greater than 24 months

Exclusion criteria:

(I) conditions precluding the implantation of a Nit-Occlud® Lê VSD coil, such as peri-membranous VSD without aneurysmal tissue
(II) associated cardiac anomalies requiring surgery
(III) active endocarditis or other active infections at time of implantation

The study has been reviewed by an independent ethics committee according to German medical device law. Furthermore, each clinical investigator did consult the relevant local ethics committee. Finally, the patient/patient's parents gave their written consent for participation.

#### Interventional procedure

**Fig. 2:**
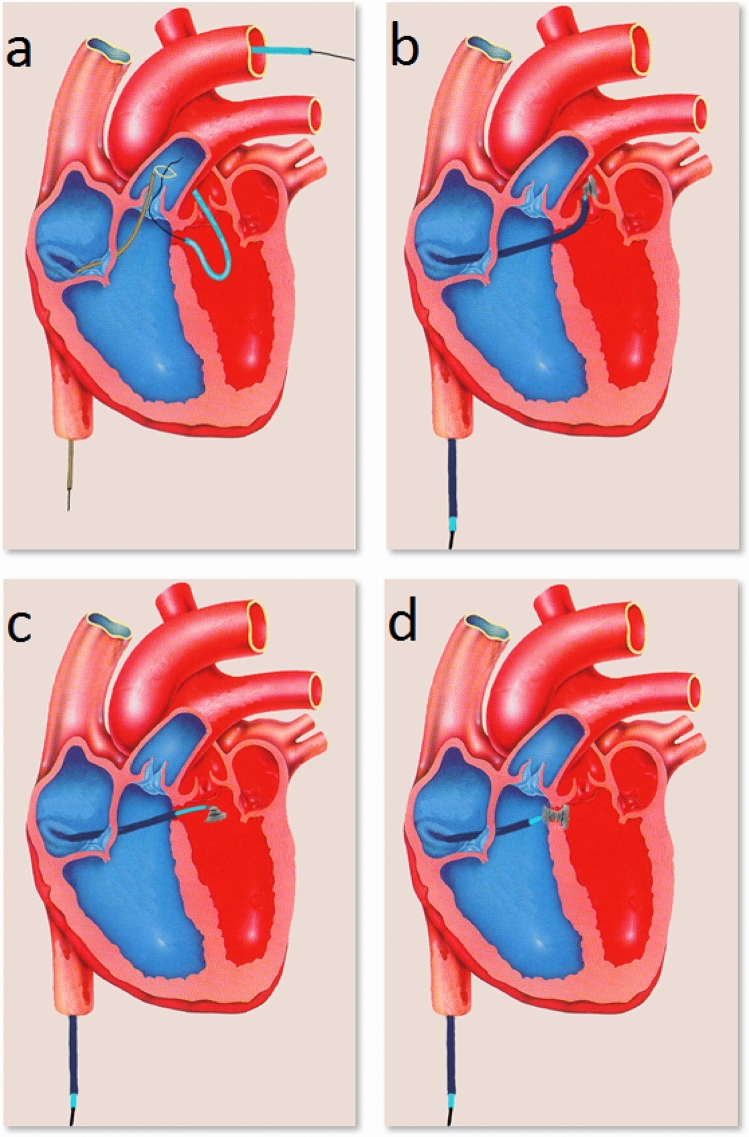
Intervention process: **a** The VSD is crossed with a wire, which is snared in the pulmonary artery to establish an arteriovenous loop, **b** positioning of a long sheath and partial configuration of the coil in the ascending aorta, **c** pull back of the coil into the left ventricular outflow tract, **d** pull back into the VSD, 1–2 loops are configured on the right side

According to the study protocol, each investigator could do the procedure under sedation or anesthesia as usual in his hospital. Left ventricular angiography in 30°–60° left anterior oblique view with cranial angulation was used for imaging the VSD ± an additional lateral view. VSD diameter was measured from the left as well as the right ventricular side. Periprocedural transesophageal echocardiography was not required according to the study protocol but was performed by some physicians. After arterial and venous introducer sheaths were inserted, an arteriovenous guidewire loop was created (Fig. 2a). A long sheath was then guided from the venous access across the VSD into the ascending aorta. In contrast to most other occluders with cage design, the left ventricular part of the coil is configurated in the ascending aorta and then pulled back into the left ventricular outflow tract (Fig. 2b/c). The left ventricular side of the coil was positioned from the LV inside of the aneurysmal tissue and then the right side of the coil was released on the right side of the aneurysmal tissue and septum. After the positioning was reviewed, the coil was detached from the carrier system. The various coil sizes and associated French sizes for the long sheath are listed in Table 1.

**Table 1:** Coil specifications and numbers of finally used devices

Coil specification	Distal coil diameter (LV)	Distal coil diameter (RV)	Recommended long sheath size	Numbers used for pVSD closure
8 × 6	8 mm	6 mm	6 F	11 (12.9%)
10 × 6	10 mm	6 mm	6 F	40 (47.1%)
12 × 6	12 mm	6 mm	6 F	21 (24.7%)
14 × 8	14 mm	8 mm	7 F	9 (10.6%)
16 × 8	16 mm	8 mm	7 F	4 (4.7%)

During the intervention, the systemic administration of 50–100 units of heparin per kg of body weight was given (activated clotting time (ACT) 200–250 s). Antithrombotic prophylaxis with acetylsalicylic acid was started immediately after device implantation and continued for 6 months.

#### Technical and clinical investigations

The study plan included recording data from the pre-implantation, immediately after the implantation, at discharge and aftercare after 2 weeks, at 3 and 12 months, and at 2 and 5 years. Echocardiographic examinations had to be performed according to a study-specific echocardiography guideline and had to be recorded to allow additional offline central analysis. An independent core laboratory validated the echocardiographic reports.

Measurements for residual shunts were done by angiography immediately after device implantation and by echocardiographic means during follow-up. The residual shunts were graduated into trivial, small and moderate. A trivial shunt was defined as an echocardiographic finding with a minimal signal in color Doppler indicating a residual shunt, but without a complete flow profile in pulsed wave/continuous wave (PW/CW) Doppler. The vena contracta of the color jet in echocardiography was defined as < 1 mm for trivial shunts, 1–1.5 mm for small shunts and > 1.5–2 mm for moderate shunts. Complete closure as well as a trivial residual shunt was defined as successful closure. A 12-lead ECG was used during each check, and Holter monitoring was used only in cases showing abnormality.

#### Classification of serious and major serious events

Device-related serious adverse events were classified as potentially life-threatening or requiring surgery to correct. These were increase in valve insufficiency by at least 2 degrees, any problem necessitating surgical explantation of the device, e.g. hemolysis, thrombus, device fracture, embolisation of the device, device-related stroke, myocardial infarction, persistent cardiac arrhythmia requiring permanent pacemaker.

Device-related major adverse events were classified as requiring medical or transcatheter intervention to correct. These were blood loss or haemolysis requiring blood transfusion, device embolisation or malposition requiring transcatheter retrieval, device-related infection requiring medical treatment, reversible neurological event, myocardial ischaemia without comprised cardiac function, vessel comprised requiring anticoagulation thrombolysis, arrhythmia or temporary heart block requiring medical treatment or temporary transvenous pacing.

### Statistical analysis

Data processing, validation, and evaluation were performed by external and independent statistical analyses which were carried out with the statistical software packages SAS® and R. All confidence intervals are calculated as two-sided intervals on the basis of a 95% confidence level. No missing data imputation was performed for the reported analyses.

### Data quality assurance

The monitoring of the German centers was performed by employees of the sponsor. In accordance with Good Clinical Practice (GCP), the sponsor had nominated a Contract Research Organisation (CRO) as external auditor for this clinical investigation in Germany as well as in Israel. The quality of the closure was checked by a non-study expert by analyzing the echocardiographic images from the study centers.

## Results

**Fig. 3:**
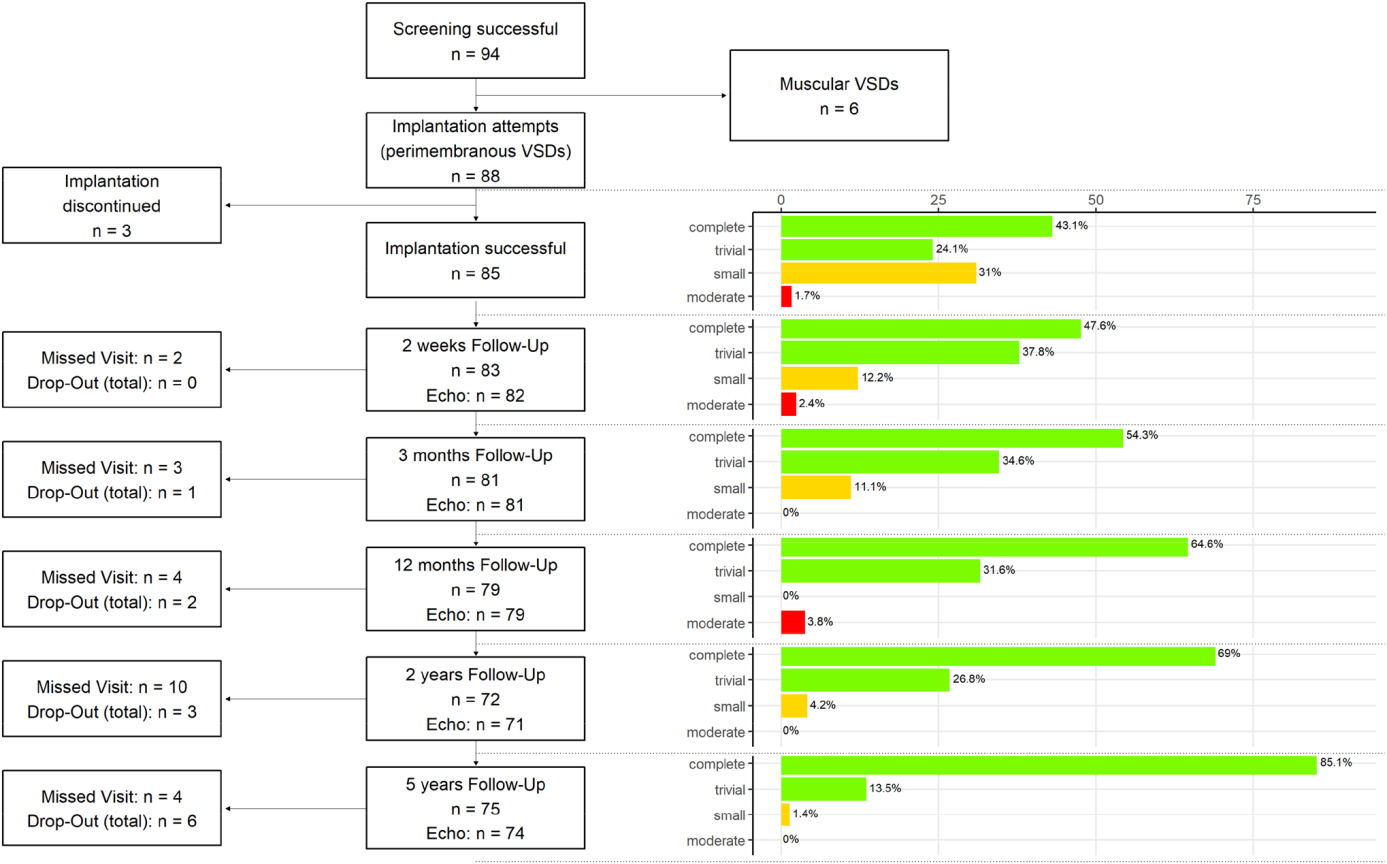
Flowchart for the study population. Closure rates as determined by echocardiography. The green columns represent the percentage of closed VSDs (first column: complete closure, second column: trivial shunts, vena contracta of color jet in echocardiography < 1 mm), yellow = small shunts (1–1.5 mm) and red = moderate shunts (> 1.5–2 mm)

From October 2006 to June 2011, 94 patients with ventricular septal defect were screened for coil closure in six tertiary centers in Germany and Israel (see flowchart, Fig. 3). Six patients were excluded from the analysis because their VSDs were classified as muscular. The demographic data of the 88 intended-to-treat patients are summarized in Table 2.

**Table 2:** Patient and VSD characteristics

	Median	(min—max)	(*n*/88)
Study population (*N* = 88; female *n* = 50 (56.8%)			
Age [years]	8	(2–65)	(88)
Weight [kg]	26.7	(10–109	(88)
Height [cm]	128.5	(82–193)	(88)
VSD diameter RV [mm]	4	(2–8)	(88)
VSD diameter LV [mm]	9	(5–19)	(85)
Distance between RV and LV opening [mm]	6.5	(3–16)	(81)
Distance between rim and aortic annulus [mm]	4	(3–10)	(82)

Implantation of the device was completed technically successfully in 85 of 88 patients, which corresponds to a technical success rate of 96.6% (confidence interval (CI): 90.4–99.3%).

In three patients, a closure of the defect could not be performed. In all of them, the anchoring of the device was not possible because of absent aneurysmal tissue or a left ventricular defect size of  > 8 mm. According to the study protocol, these patients were excluded, and no further data were recorded. According to the last information provided by the investigators, one patient received a surgical VSD closure, another also after the defect could also not be closed with an Amplatzer occluder. There is no information about the third patient.

In 71 patients (80.7%), the VSD was closed in the first attempt, in 11 patients (12.5%), a second, and in 3 (3.4%), a third attempt with a different coil was needed for closure. Mean procedure time was 46.6 (± 23.4) minutes (min), and mean fluoroscopy time was 25.2 (± 12.8) min. The hospital stays ranged from 1 to 10 days with a mean of 2.9 (± 1.2) days. The echocardiographic measured values for the left and right ventricles were assessed normalized to the body surface. Left ventricular volume loading (> 2 standard deviations than normal) was one of the required inclusion criteria. At the last follow-up, all parameters of the left and right heart were within the normal range.

### Closure rates

The closure rate was 67.2% immediately after the interventional procedure and increased to 86.2, 89.5, 96.4 and 98.7% after 2 weeks, 3 months, 12 months and 5 years after implantation, resp. (Fig. 3). In three patients, a moderate shunt persisted up to 12 months and resolved after 5 years.

12-month follow-up data were obtained in 79 patients, while 10 were missing the five-year follow-up examination (Fig. 3).

### Device or procedure-related serious adverse events

Three serious adverse events occurred during follow-up after device implantation corresponding to a rate of 3.5%. Hemodynamically relevant tricuspid valve regurgitation occurred in two patients after device implantation. Since there was no improvement after several months, the valves were reconstructed surgically. In one of these patients, the device was explanted during the procedure. The further outcome was uneventful in both patients.

In one patient, during routine examination after four months, a vegetation on the device was revealed by echocardiography [17]. Kingella kingae was isolated from blood cultures. Because of the very large and unstable vegetation, the coil and the vegetation were removed surgically, and the VSD was closed with a pericardial patch. After 6 weeks of parenteral antibiotic therapy, the postoperative course was uneventful.

No further serious adverse events or deaths were experienced during the study period.

### Device or procedure-related major adverse events

The study results exhibit a major adverse event rate of 5.6% (5 events in 85 patients). In two patients, the device embolized during implantation. One embolization was caused by a sizing error, the other by a premature release of the device within the catheter. In both cases, the device was retrieved with a snare and the VSD was subsequently successfully closed with another coil of different size.

Complications at the arterial access site occurred in two patients which were successfully treated with systemic heparin infusion and fibrinolysis.

In a patient with two closely adjacent ventricular septal defects, hemolysis with a fall in hemoglobin levels occurred a day after implantation. Since hemolysis was most likely caused by the jet flow of the second defect through some overlapping loops of the coil, the second VSD was closed with an Amplatzer ADO II device which covered this area, and the hemolysis subsequently ceased.

### Device or procedure-related minor adverse events

Minor hemolysis (9.4%) was seen in eight patients after implantation and lasted for a period of one to 14 days. It resolved spontaneously in all patients.

In nine patients, self-limiting rhythm disturbances were observed after coil implantation, 1st-degree atrio-ventricular (AV) block (*n* = 3), an intermittent AV rhythm or sinus bradycardia in five patients, and premature ventricular beats in one patient which resolved spontaneously during the procedure. There were no acute or late AV blocks of 2nd or 3rd degree. Fractures and embolization of parts of the coil did not occur in the long term.

## Discussion

Despite the introduction of transcatheter closure of perimembranous and muscular ventricular septal defects in 1998 and the use of different devices since the early 2000s, surgery remains the standard treatment for the peri-membranous VSD [1, 7].

When compared with surgical results, the initially relatively high incidence of complete AV block from the transcatheter approach for perimembranous VSDs has been a cause of great concern [2]. It has been suspected that device oversizing, deployment of the device in close vicinity to the bundle of His and the clamping of the interventricular septum by the double-disc devices are causative [27].

In contrast to the available “stenting” VSD devices, the Nit-Occlud® Lê VSD device was introduced as a flexible coil device, which adjusts itself to the structures of the heart, thereby potentially avoiding the high incidence of complete AV block.

In the presented study, a total of 94 patients with VSD from six centers in Germany and Israel were screened with the intention of transcatheter/interventional closure using the Nit-Occlud® Lê VSD coil. The device was implanted successfully in 85 of the 88 intent-to-treat patients, which gives a technical success rate of 96.6%. The main reason for failure was inadequate periprocedural sizing of the ventricular septal defect. A similar success rate of VSD closure using the Nit-Occlud® Lê VSD coil has been described in later studies [11, 25]. These patients were followed over a 3-year period in 18 European centers. Definitive device implantation was successful in 91.9% patients. This was the initial study for introducing this device in clinical practice and, therefore, incorporates the learning curve for the operators using this coil. The final closure rates are close to those reported by others [6, 9, 16, 20, 21, 23, 25, 29].

The data presented here show a clinical success rate of defect closure in 67.2% immediately after implantation, based on angiographic imaging. During follow-up, the rate increased to 96.4% and 98.7% within 12 months and 5 years, respectively, evaluated by echocardiography. The improvement of the occlusion process with time is well explained by neo-endothelization caused by a time-dependent maturation pattern of the fibroblast-like cells in the neotissue around the implants [8]. At the 12-month follow-up, 3 patients were identified with moderate residual shunt. In this trial, the responsible interventionalists decided to give them some more time. After 5 years and with no further intervention, two of the remaining shunts were completely closed, and one was classified as small. None of the patients had any enlargement of the left atrium or left ventricle in the long-term follow-up.

Concerning the adverse events, there was no transient or permanent AV-block III^°^ during or after implantation in our cohort. This finding corresponds to those described in the post-admission trial EUROVECO-Registry [11] and stands in contrast to the Amplatzer registry with an AV block rate of 5.7% [2–4]. Even with the so-called second-generation devices, AV block could not be completely eliminated [10, 20]. Recently, it was shown that with new devices, the long-term complication rate is low, however, eccentric, large devices, and long fluoroscopy time increase the risk of early postprocedural arrhythmias after transcatheter closure of VSDs up to 24.1% [29]. However, a study from Egypt showed that the coil can also cause an AV block and they had to abandon the procedure for this reason in one case [25]. The pfm occluder has a smooth device design and should be located within the aneurysmal formation of the VSD to make an AV-block III^°^ unlikely. However, the three cases of AV-block I^°^ in our series might indicate that there is still a potential risk. Double-umbrella devices are additionally used in VSD without aneurysmal formation and due to the clipping and stenting mechanism, the risk of causing arrhythmias for these types of defects is potentially higher. Nygyen showed that percutaneous pVSD closure using either Nit‐Occlud Lê VSD Coil or Amplatzer Duct Occluders is feasible, safe and efficacious in selected patients [21]. The main problems of Duct Occluders are unsuitable defect anatomy and device embolization, while VSD Coil disadvantages are residual shunt and hemolysis.

Tricuspid valve regurgitation occurred in two patients after device implantation in our study. In comparison, a mild tricuspid regurgitation was observed in five patients in the EUROVECO-Registry [11]. It can be assumed that severe tricuspid valve incompetence might be caused by positioning the sheath through the chordae of the valve or by entangling the chordae with the coil itself. This complication might be avoided by careful periinterventional echocardiographic guidance and adequate catheter choice and handling. Tricuspid regurgitation was also an issue in the Amplatzer registry [2, 4, 13]. Yang et al. in their systematic review of 37 publications on 4406 patients with closure of VSD with different devices from 2003 to 2012 found a rate of 1.7% hemodynamically relevant tricuspid insufficiency [28].

Device embolization occurred in two of our patients (2.4%) and could be easily resolved by retrieving the devices with a snare. The reason for this complication usually is incorrect sizing of the VSD, which is critical for the selection of the coil. In the review of Yang, embolization was seen in 0.4% [28]. In this context, the use of transesophageal echocardiography is generally recommended to optimize information on shape and size of the defect.

The most commonly discussed issue of interventional VSD closure is the residual shunt, which usually is of limited duration. This may cause hemolysis up to the point of complete closure. In this study, post-interventional hemolysis of minor degree occurred in eight patients; it resolved during the first few days after implantation without further intervention. Only in one patient with two ventricular septal defects did severe hemolysis occur after closure of one defect and resolve with device closure of the second VSD (1.2%). Hemolysis following implantation of the Nit-Occlud® Lê VSD coil as well of the Amplatzer VSD device or other mesh devices has been described by others at a rate of around 2% [11, 13, 22]. The immediate residual shunt was shown as marginally higher for the Nit-Occlud® Lê VSD coil when compared with the Amplatzer VSD device [5]. In the EUROVECO-study with 102 patients, large residual shunts and severe hemolysis required explantation of the device in two patients (1.8%) resulting in prompt cessation of the hemolysis [11]. The dimensions of the residual shunt and the degree of hemolysis appear to be correlated. A recent study on the initial experience in France with the Nit-Occlud® Lê VSD coil showed a higher rate of severe hemolysis in 8 of 46 cases in which 4 cases needed further invasive therapy [14]. The longer than average hospital stays in our trial compared to other studies were due to longer monitoring times in patients diagnosed with initial moderate hemolysis.

## Conclusion

This multicenter, prospective study demonstrates a high long-term success rate of VSD closure with the pfm Nit-Occlud® Lê VSD device in aneurysmal perimembranous VSD. In addition, the reported results have shown that the Nit Occlud® Lê VSD coil is a safe alternative to other available devices with a comparatively acceptable rate of serious adverse events. The coil device seems to lead to fewer rhythm complications but has an initially higher rate of residual shunts with the risk of hemolysis. Minor hemolysis usually ceases spontaneously within a short time period. With a significant residual shunt, hemolysis still remains a concern and seems to be associated with the shunt. Therefore, the Nit-Occlud® Lê VSD coil offers the possibility for an effective and safe approach in patients with aneurysmal perimembranous ventricular septal defects. Further studies are necessary to compare the Nit-Occlud® Lê VSD coil system with other conventionally used devices, such as Amplatzer devices.

### Study limitations

The intended sample size of this prospective study was limited due to a change in European and German law while recruiting patients for the study. Because of the fact that all data collected up to that time met the requirements for approval in Europe, it was decided to follow the long-term course of already enrolled patients and to discontinue the recruitment of further patients. Due to limitations in the sample size of this prospective study and changes in the European law, it is necessary to conduct further multicenter studies to verify the occurrence rates of residual shunts and hemolysis of this device.

The reduction in the number of follow-up examinations was attributed to the fact that some patients or parents did not want to continue the examination, especially if the VSD was already completely closed.
